# Hypodermic Injections

**Published:** 1869-11

**Authors:** H. C. Smith

**Affiliations:** Millville N. J.


Hypodermic Injections.—Much has been said of late in reference to the hypodermic syringe in various painful affections, of its great success in the relief of suffering, and as well as the ease and facility with which it may be employed. I have never used it till within the last few weeks, but have already learned to value it as one of my most useful and reliable agents. The following case may be of some service to such of my professional brethren, who like myself, have known it only by hear-say. This, with the fact that I have never read of its employment in a similar case, is my excuse for sending this article.

On the morning of October 31st, Drs. Whitaker, Newell, and myself, were called to attend Michael Stokes, who had been run over by a steam fire engine. We found our patient in a state bordering on collapse, and after ordering stimulants had him removed to his residence. After removing his clothing, and making a careful examination, we found an oblique fracture of the right femur, with a comminuted fracture of the lower extremity of the tibia of the same side. The tibia and fibula of the left limb were fractured transversely in the upper third.

In reducing this complicated injury it was necessary to take some measure for the prevention of the acute suffering which would naturally accompany our manipulation. His physical condition rendered chloroform inadmissible, and we had recourse to hypodermic use of sulphate of morphia. We accordingly injected gr. ¼ in the flesh part of the *fore-arm*; the effect was seen in three minutes, when we reduced the fracture and applied the splints to the right limb without any complaint on the part of the patient. Another gr. ¼ was then injected with the same happy result, the two doses being sufficient to keep him quiet during the whole dressing, which required some three hours.

The important points, to my mind. are:

1st. We were enabled to remove the pain, when chloroform was contraindicated. 2nd. The amount used was not more than one-fourth of what would have been required if administered by the mouth, and the effect was almost immediate. 3d. Any anæsthetic, properly so called, would have required a special assistant for its administration and control.

I think that the above case shows that the "syringe" may be used with advantage in cases of fracture and kindred injuries, where chloroform is either inconvenient or contraindicated.

*Millville, N. J.* H. C. SMITH, M. D.

